# Altered muscle activity during rest and during mental or physical activity is not a trait symptom of migraine - a neck muscle EMG study

**DOI:** 10.1186/s10194-018-0851-5

**Published:** 2018-03-20

**Authors:** Kerstin Luedtke, Jan Mehnert, Arne May

**Affiliations:** 1 Department of Systems Neuroscience, University Medical Center Eppendorf, Martinistraße 52, 20246 Hamburg, Germany; 2 Department of Academic Physiotherapy, University of Luebeck, Luebeck, Germany

**Keywords:** Migraine, Trigger, Neck pain, Muscle tension, EMG, Stress

## Abstract

**Background:**

Migraineurs have a high prevalence of neck pain prior to or during headache attacks. Whether neck pain is a symptom of migraine or an indicator for a constant neck muscle dysfunction potentially triggering migraine attacks is a topic of scientific debate. The presence of myofascial trigger points in neck muscles including the trapezius muscle, points towards muscle alterations associated with migraine. We measured electromyography (EMG) of the neck muscles in a large cohort to identify whether neck pain and neckmuscle tension reported by migraine patients can be attributed to increased neck muscle activation during rest, mental stress or physical activity.

**Methods:**

Surface EMG responses of the trapezius muscle were recorded during a paradigm including rest periods, mental stress and physical activity of 102 participants (31 chronic migraine, 43 episodic migraine, 28 healthy participants).

**Results:**

All groups showed increased trapezius activity during mental stress and physical activity compared to rest. There was no statistically significant difference between migraine patients and healthy controls for any of the 3 conditions except for the initial mental stress situation (F (2,56.022) = 8.302, *p* = 0.001), where controls increased tension by only 4.75%, episodic migraineurs by 17.39% and chronic migraineurs by 28.61%. Both migraine groups returned to resting EMG levels within the same timeframe as healthy controls.

**Conclusions:**

Neck pain associated with migraine can therefore not be attributed to increased trapezius activity during rest, mental stress and physical activity or prolonged muscle activity and should not be seen as a constantly underlying trigger but rather as an accompanying symptom of migraine.

## Background

The high prevalence of neck pain in patients with migraine [1–3] perpetuates an ongoing debate about the contribution of neck muscles to headache provocation and the frequency of attacks. While some authors claim that neck pain is simply a symptom of the migraine attack [2], others postulate that dysfunctions in neck muscles may trigger migraine attacks and thereby contribute to the transition from episodic to chronic migraine [4].

The latter hypothesis is based on publications that identified a higher prevalence of active and latent trigger points in neck muscles of patients with migraine compared to headache-free control populations [4–6]. This point of view is further supported by a study with a local anaesthetic (Ropivacaine) injection into these trigger points, which effectively reduced migraine frequency in approximately 60% of the participants in a non-controlled design including 52 chronic migraine patients [7] and another one which suggested beneficial effects of local therapy of active myofascial trigger points [8].

Other publications focusing on muscle tension in headache populations used surface electromyography (EMG) recordings either during rest or during physical or mental tasks. While higher levels of muscle tension, especially as a response to stressful mental tasks [9, 10], or to painful stimulation [11], are consistently found in patients with chronic tension-type headache (TTH), the evidence in migraine is conflicting: Sandrini et al. (1994) did not find altered EMG recordings from the frontalis muscle during mental arithmetic in migraine patients [10]. This was later confirmed by Leistad et al. (2006) who only found differences in trapezius muscle responses to 60-min periods of cognitive stress in patients with TTH but not in patients with migraine or in healthy controls [12]. Goudswaard and colleagues [13] reported that migraine patients consistently showed higher proportional EMG levels of the corrugator muscle during periods of experimental and real-life stress but patients who developed headaches during stress had lower levels of muscle tension than participants without headaches. The authors concluded that increased EMG activity during stress seems not to be the cause but rather a response to head pain [13]. A similar observation was reported by Pritchard and colleagues [14], who found that the occipital muscle output during an experimental stressor was significantly lower in the migraine group and higher in the tension-type group during the headache phase [14].

Opposing results were reported by van Boxtel et al. (1983) who recorded significantly higher EMG levels in the migraine and mixed headache groups when expressing the resting EMG levels as a percentage of the EMG level during maximal contraction [15]. In girls with “migraine-type” headache, a higher EMG/force ratio between the EMG of the left agonist sternocleidomastoid (SCM) muscle and the corresponding maximal neck flexion (*p* = 0.030) was observed [16]. Furthermore, during a physical task, a higher co-activation of the superficial extensor muscles (trapezius) was recorded in chronic migraineurs compared to healthy controls [17].

Interestingly, Leistad and colleagues (2006) reported a delayed recovery of migraine patients and controls from a painful stimulus as well as a delayed return of trapezius EMG activity to baseline, which was only significant in tension-type headaches but also more prolonged in migraine compared to healthy controls [12].

Neck muscle relaxation techniques including EMG biofeedback are frequently used as non-pharmacological additions to reduce the frequency of migraine attacks and are consequently recommended by national and international guidelines [18–20]. In a review, all relaxation methods including EMG, achieved an average reduction in migraine frequency of 35–45% [21], although blood-volume-pulse feedback yielded higher effect sizes than peripheral skin temperature feedback and electromyography feedback [22]. However, in a clinical trial, EMG biofeedback training had no additional effect over education [23].

The inconsistency of the existing evidence and two important additional questions triggered the design of the current study: question 1 is, whether activities of remote body parts, e.g. the lower limb will result in a higher activation of neck muscles in migraine patients, and question 2 is, whether neck muscle tension is prolonged after any type of stress or physical activity.

Therefore, the purpose of this study was to evaluate the level of muscle tension during voluntary relaxation and during mental and physical tasks as well as the ability to relax after periods of mental and physical stress in patients with migraine and headache-free control participants. The question was, whether migraine patients exhibit a constantly raised muscle tone or, if not, whether migraineurs are more susceptible to increased neck muscle tension under stress (mental of physical) which theoretically could also suggest that muscle tension is a trigger to induce migraine attacks.

- Hypothesis 1: The mean EMG response for both, physical and mental stress differs between healthy controls and patients.
- Hypothesis 2: Migraineurs’ neck muscles need more time to recover from mental and physical tasks.

## Methods

### Subjects

There were no adequate previous publications to base a sample size calculation on. The difference between migraine patients and controls was anticipated to be rather large considering that neck pain is such a dominant symptom in migraineurs. A sample size of 102 was sufficient to detect an effect size (Cohen’s d) of 0.4 with 95% power and an alpha level of 0.05 [24]. All consecutive patients attending a headache outpatient clinic in Germany that were diagnosed with migraine (with or without aura) according to the criteria published by the international headache society [25], by a neurologist specialized in the assessment and treatment of headache patients, were invited to participate. They received an information leaflet stating the inclusion/exclusion criteria and the data collection procedure in detail. Of those, who responded to this invitation, none had to be excluded.

Control participants were recruited using a university platform, social media and personal contacts, that had a maximum of 3 headache attacks per year that did not fulfil the criteria of migraine or any other primary headache type except sporadic episodic tension-type headache. For all groups, the following exclusion criteria were applied: not younger than 18 years, not suffering from a neurological, psychiatric or pain condition (other than migraine) as well as rheumatoid arthritis or diagnosed neck pathology (such as whiplash associated disorder or disc disease). Migraine patients were excluded if they took pain medication on more than 10 days in the past month or suffered from any relevant additional headache type.

After providing written informed consent, all patients were asked to fill out questionnaires regarding their quality of life reduced by headache (MIDAS [26], or neck dysfunctions (NDI [27], and the Patient Health Questionnaire (PHQ [28]). Furthermore, we assessed whether they had headache at the day of the examination, when the last and the next headache attack was, how many years of headache they had in their history, the number of days per month suffering from headache as well as the dominant headache side.

### Experimental design

The experiment consisted of 7 experimental conditions alternated with relaxation periods, resulting in a total of 15 blocks. Three blocks were mentally stressful: participants had to count backwards from 100 in steps of 9 (from 80 in steps of 7 and from 60 in steps of 4, respectively for 3rd and 6th block) as fast as possible, while the other 4 conditions included physical lower limb activity: either pressing 1 foot down on a body weight scale and holding a minimum of 27 kg or resisted knee extension of one leg for 30 s. Tests were performed with each leg separately. At the start and end of the experiment further 2 min relaxation blocks were added. An overview of the experimental timeline can be found in Table 1.

**Table 1:** Experimental design

Block	Condition	Approx. duration [sec]
1	rest	120
2	counting backwards in steps of 9	45
3	Rest	60
4	counting backwards in steps of 7	45
5	Rest	60
6	counting backwards in steps of 4	35
7	rest	60
8	scale right leg	30
9	rest	60
10	scale left leg	30
11	rest	60
12	knee extension right	30
13	rest	60
14	knee extension left	30
15	rest	120

### EMG data acquisition and pre-processing

During the experiment, electromyography was measured with two channels located at both sides of the neck over the upper trapezius muscle with a distance between electrodes of 20 cms. Surface EMG was recorded using a Schuhfried Biofeedback x-pert 2000 system (Dr. Schuhfried Medizintechnik GmbH, Wien, Austria) with a sampling frequency of 500 Hz in the frequency band of 100 to 200 Hz to measure muscle activity and a measuring range of 60 μV (resolution: 0.048 μV). A digital notch filter attenuated power line noise at 50 Hz. This system provides voltages for a moving temporal integral (250 ms) of absolute summed action potentials.

For further processing by self-written routines using the software Matlab (Mathworks, Version R2014a), data was down-sampled to 10 Hz and high frequency noise was reduced by 3 iterations of outlier reduction, where in a moving window of 20 sampling points values exceeding 4 times the standard deviation were replaced by the windows’ mean. Sharp transients and ECG artifacts were eliminated with a median moving-window filter (filter order 500, build-in medfilt1 function in Matlab). Afterwards, data was segmented into the epochs of the experimental conditions described above (Table 1).

### Data analysis

The person analyzing all data was blinded to the diagnosis. Differences across groups regarding migraine disability (MIDAS), depression (PHQ) and neck disability (NDI) were calculated using the Mann-Whitney-U test as a non-parametric option for not normally distributed data [29]). To test hypothesis 1 we calculated the proportion (percentage) of the difference to the previous relaxation block’s mean for each of the 7 experimental conditions of the EMG signal. We tested the main effect of mental or physical stimulation with independent, one-sample t-tests and used one-way-ANOVAs to assess significant differences between the three groups for each condition separately. Whether the presumption of homoscedasticity was fulfilled was tested using Levene’s test. Whenever this test was significant, Welch’s test was used. To correct for multiple testing, Bonferroni correction was applied. Any significant difference was further tested for a relation to the side of headache. We therefore used point-biserial correlation as a special case of the Pearson’s correlation to identify a correlation between the dominant headache side and the tested side of the neck. Subjects with bilateral headache were analyzed in both groups of dominant headache sides. As expected when including chronic migraine patients, a significant number of participating patients reported headache during the examination we further ran a two-sample t-test for all 7 blocks and 2 EMG channels to investigate the influence of acute headache on neck muscles tension. To further investigate an influence of the preictal phase we run two-sample t-tests between patients with headache in a 24 h (48 h, 72 h, respectively) period before the examination against patients which were headache free for the minimum of 4 days.

To test hypothesis 2 we calculated the time until the mean value within the relaxation block was reached after a mental or physical task and calculated a one-way-ANOVA between the three groups for each condition separately. All statistical analyses were performed using either Matlab (MathWorks, Version 2014a) or SPSS (IBM) Version 24.

## Results

### Clinical data

A total of 102 volunteers participated in the study between June 2016 and May 2107. Twenty eight were headache-free control participants (21 females; mean age 43.3 years (Standard deviation (SD) 12.2), min: 23 years, max: 70 years). Forty three subjects suffered from episodic migraine (38 females; mean age 39.12 years (SD 12.2), min: 18 years, max: 60 years), i.e. had headache on fewer than 14 days per month (mean 7.3 days, SD 3.6), and 31 patients were diagnosed with chronic migraine (30 females; mean age 39.3 years (SD 13.8), min: 20 years, max: 68 years) with 15 or more days of headache per month (mean: 21.7 days, SD: 5.6). Age was not significantly different between the three groups (*p* > 0.3, F (2,98) = 1.065, One-Way-ANOVA).

MIDAS, NDI and PHQ scores were significantly different between patient groups (*p* = 0.02, *p* = 0.01, and *p* = 0.048, respectively) with chronic patients suffering more (see Table 2).

**Table 2:** Clinical characteristics of participants

	Control (*n*=28)	Episodic migraine (*n*=43)	Chronic migraine (*n*=31)
Age [mean (SD)/range]	43.3 (12.2)/23-70	39.12 (12.2)/18-60	39.3 (13.8)/20-68
Gender [female / male]	21 / 7	38 / 5	30 / 1
Duration of disease (years) [mean (SD)]		17.3 (10.7)	15.0 (15.3)
Intensity (0-10) [mean (SD)]		6.5 (1.6)	6.9 (1.7)
Frequency (days per month) [mean (SD)]		7 (4)	22 (5)
MIDAS [mean (SD)]		35.8 (31.4)	68.5 (61.6)
NDI [mean (SD)]	3.0 (3.1)	12.0 (6.1)	16.6 (6.1)
PHQ [mean (SD)]	3.7 (3.9)	6.9 (3.8)	8.7 (4.4)
Days since last headache [mean (SD)]		4 (6)	2 (3)
Days until next headache [mean (SD)]		5 (8)	1 (2)

*MIDAS* migraine disability assessment test, *NDI* neck disability index, *PHQ* patient health questionnaire, *SD* standard deviation

Thirteen of the episodic patients (31.7%, 2 values missing) and 20 of the chronic patients (64.5%, 2 values missing) reported headache during the examination. Furthermore, 24 episodic (58.5%) and 24 (82.8%) chronic patients had headache within the 48 h before the examination and 21 (56.8%) episodic and 23 (85.2%) chronic patients had headache within 48 h after the examination.

### EMG

**Fig. 1:**
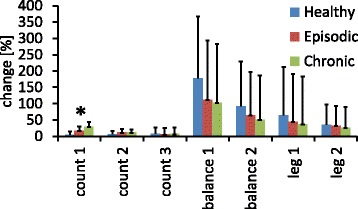
EMG changes relative to rest blocks in the electrode over the right neck muscle for the three individual groups and each experimental condition. The errorbars represent standard deviations. The asterisk marks a significant group difference (*p* < 0.05)

All groups showed increased bilateral trapezius muscle activity during mental as well as physical stress (for all tasks *p* < 0.05). There was no difference across groups for the mean EMG increase (relative to the previous relaxation block) during any of the tasks apart from the first mental stress task which showed significantly higher EMG activity on the right side during counting backwards in steps of 9 (Fig. 1). Levene’s test for homoscedasticity was significant, therefore Welch’s Test was used (F (2,56.022) = 8.302, *p* = 0.001). The mean EMG amplitude change was 4.75% (SD 10.29%) for the healthy subjects, 17.39% (SD 25.34%) in the episodic migraine group, and 28.61% (SD 40.12%) in the chronic migraine group in the electrode covering the right neck muscle. The difference between the two migraine groups was not statistically significant in the Bonferroni corrected post-hoc testing (*p* = 0.28) and neither the difference between healthy controls and episodic migraineurs (*p* = 0,20), Nevertheless, the comparison between healthy controls and chronic migraineurs yielded Bonferroni corrected significance (Bonferroni, *p* = 0.005). There was no significant (point-biserial) correlation between the dominant headache side and the recorded side of the neck. Furthermore, we could not find any significant differences between patients with and without acute headache during the examination (*p* > 0.05 in all two-sampled t-test) as well as between preictal patients (in a 24 h, 48 h as well as 72 h period) and patients which were free of headache since at least 4 days.

**Fig. 2:**
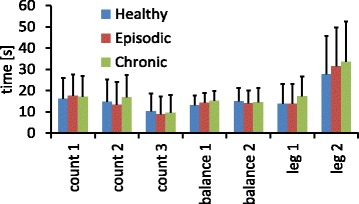
Duration until the average relaxation value within the resting blocks was reached in the electrode over the right neck muscle for the three individual groups and each experimental condition. The errorbars represent standard deviations

The second hypothesis, that the time required to relax after a stressful task differs between migraine patients and healthy controls, had to be rejected, because there was no significant difference across groups for any of the relaxing periods as tested by a one-way ANOVA (Fig. 2).

## Discussion

The migraine population did not differ from healthy participants regarding EMG-recorded muscle tension of the upper trapezius during mental and physical tasks, except for the first task (counting backwards from 100 in steps of 9). Furthermore, the results did not indicate a delayed recovery of trapezius EMG activity from mental or physical tasks, as previously reported for tension-type headache [12]. This unanticipated result suggests that altered muscle activity during rest or during mental or physical activity is not a trait symptom of migraine, in contrast to tension-type headache [9, 30–32]. Hence, the frequently observed neck pain before or during the migraine attack [1–3] is more likely a symptom of the migraine attack and less likely an indicator for a neck muscle pathology that subsequently triggers an attack. Headache status on the day of testing did not significantly influence the results, therefore neck pain or perceived muscle tension does not seem to be quantifiable with surface EMG data.

It further suggests, that the migraine reducing effect of neck muscle EMG-biofeedback is probably not attributed to a reduction in muscle tension but to a general relaxation effect, further supported by Nestoriuc and Martin (2007) who reported that blood-volume-pulse feedback yielded higher effect sizes than peripheral skin temperature feedback or EMG feedback [22]. Our results are in line with the results from Leistad et al. (2006) who used a slightly different paradigm (60 min of cognitive stress preceding 30 min of relaxation) but reported no difference regarding EMG recordings from the neck muscles between migraine patients and controls [12]. Furthermore, Sandrini and colleagues also applied EMG measurements as a diagnostic tool and reported significantly altered muscle activity in patients with tension-type headache but not in patients with migraine [10]. The higher EMG levels reported by van Boxtel and colleagues refer to the temporalis muscle and not to muscles in the neck [15].

Significantly increased trapezius muscle tension in chronic migraine patients compared to controls during a physical task was reported by Florencio et al. (2016). Participants were required to perform a test aiming to measure deep cervical flexor activity. During this task chronic migraine patients showed significantly more co-activation of the extensor muscles than controls and episodic migraine patients. The authors explain their findings with pain-induced alterations of motor patterns [17]. While this might be a motor strategy for localized tasks such as upper cervical flexion, our findings reveal that altered motor patterns are not generalizable to activities of the lower limb, indicating that there is no tendency towards increased neck muscle tension in situations that do not require neck muscle strength.

One of the limitations of this study was that only one muscle was tested, hence not allowing to compare neck muscle tension with tension in e.g. the jaw muscles. Furthermore, EMG recordings were standardized using voluntary relaxation rather than contraction. And surface EMG recordings are not as precise as needle EMG recordings, which, however, would not have been feasible in our trial. An additional limitation is that headache assessment was based on history and not on headache diaries. However, patients were recruited at a specialised headache clinic. Most of the participants had used diaries for several month prior to participation, hence headache frequency was established based not only on recollection.

Our results from this study neither indicate higher levels of neck muscle tension nor increased durations of tension after stressful incidents in patients with frequent episodic or chronic migraine compared to headache-free controls. Perceived neck muscle tension during the premonitory phase or accompanying migraine headache is therefore more likely a subjective perception not quantifiable by EMG and should be regarded as a symptom of the attack and not as a trapezius muscle dysfunction triggering an attack. This conclusion was based on the evaluation of the trapezius muscle; whether it can be generalized to all neck muscles remains to be investigated by future studies.

## Abbreviations

EMG: Electromyography
NDI: Neck disability index
PHQ: Patient health questionnaire
SCM: Sternocleidomastoid
SD: Standard deviation
TTH: Tension type headache
